# Assessing clinical applicability of COVID-19 detection in chest radiography with deep learning

**DOI:** 10.1038/s41598-022-10568-3

**Published:** 2022-04-21

**Authors:** João Pedrosa, Guilherme Aresta, Carlos Ferreira, Catarina Carvalho, Joana Silva, Pedro Sousa, Lucas Ribeiro, Ana Maria Mendonça, Aurélio Campilho

**Affiliations:** 1 Institute for Systems and Computer Engineering, Technology and Science (INESC TEC), Porto, Portugal; 2 Faculty of Engineering of the University of Porto (FEUP), Porto, Portugal; 3 Administração Regional De Saúde Do Norte (ARSN), Porto, Portugal; 4 Instituto Português de Oncologia do Porto Francisco Gentil (IPO-PORTO), Porto, Portugal; 5 Centro Hospitalar de Vila Nova de Gaia/Espinho, Vila Nova de Gaia, Portugal

**Keywords:** Image processing, Machine learning, Viral infection, Biomedical engineering

## Abstract

The coronavirus disease 2019 (COVID-19) pandemic has impacted healthcare systems across the world. Chest radiography (CXR) can be used as a complementary method for diagnosing/following COVID-19 patients. However, experience level and workload of technicians and radiologists may affect the decision process. Recent studies suggest that deep learning can be used to assess CXRs, providing an important second opinion for radiologists and technicians in the decision process, and super-human performance in detection of COVID-19 has been reported in multiple studies. In this study, the clinical applicability of deep learning systems for COVID-19 screening was assessed by testing the performance of deep learning systems for the detection of COVID-19. Specifically, four datasets were used: (1) a collection of multiple public datasets (284.793 CXRs); (2) BIMCV dataset (16.631 CXRs); (3) COVIDGR (852 CXRs) and 4) a private dataset (6.361 CXRs). All datasets were collected retrospectively and consist of only frontal CXR views. A ResNet-18 was trained on each of the datasets for the detection of COVID-19. It is shown that a high dataset bias was present, leading to high performance in intradataset train-test scenarios (area under the curve 0.55–0.84 on the collection of public datasets). Significantly lower performances were obtained in interdataset train-test scenarios however (area under the curve > 0.98). A subset of the data was then assessed by radiologists for comparison to the automatic systems. Finetuning with radiologist annotations significantly increased performance across datasets (area under the curve 0.61–0.88) and improved the attention on clinical findings in positive COVID-19 CXRs. Nevertheless, tests on CXRs from different hospital services indicate that the screening performance of CXR and automatic systems is limited (area under the curve < 0.6 on emergency service CXRs). However, COVID-19 manifestations can be accurately detected when present, motivating the use of these tools for evaluating disease progression on mild to severe COVID-19 patients.

## Introduction

First identified in late 2019, the coronavirus disease 2019 (COVID-19) is caused by the severe acute respiratory syndrome coronavirus 2 (SARS-CoV-2). While the majority of cases causes only mild symptoms, COVID-19 can cause difficulty breathing, pneumonia, acute respiratory distress syndrome (ARDS) and ultimately death. The SARS-CoV-2 can be easily transmitted, which makes the identification of infected individuals of the utmost importance in the containment of the pandemic^1^. Reverse transcription polymerase chain reaction (RT-PCR) is the reference standard method in COVID-19 diagnosis but can present significant turnaround time and remains subject to potential shortage. Lateral flow tests (LFT), which allow much faster turnaround time, suffer however from limited and highly variable sensitivities (38.32–99.19%)^2^. Furthermore, the development of new variants of SARS-CoV-2 can have an impact on the sensitivity of both RT-PCR and LFTs^3^.

Given the involvement of the respiratory airways in COVID-19, chest radiography (CXR) was initially proposed as an alternative screening method. While there is no single radiological feature that is indicative of COVID-19, changes include ground glass, coarse horizontal linear opacities and consolidation, most often with bilateral involvement^4^. However, most COVID-19 patients do not develop pneumonia and present a normal CXR, which significantly lowers the screening value of CXR^5^. Nevertheless, it has been proposed that people with severe respiratory symptoms could be quickly screened with CXR to distinguish between COVID-19 and other pathologies^6^. Considering that COVID-19 manifestations on CXR can often be subtle, significant experience is needed for an accurate reading of these images. Nevertheless, a high proportion of CXRs are read by technical staff, rather than by experienced radiologists^7^, and the pandemic has likely worsened this issue due to staff shortages and an even higher numbers of radiological exams. Automated image analysis for the detection of CXR radiological features could thus play a significant role in providing a 2nd opinion to support clinical decisions in the triage of COVID-19 patients.

### Automatic COVID-19 diagnosis in chest radiography

**Figure 1:**
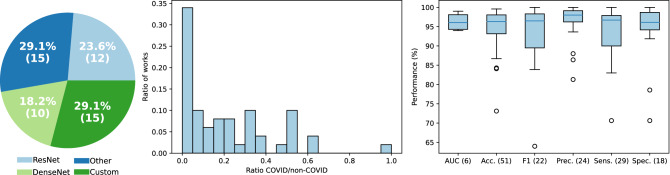
Summary of the types of deep learning architecture (left), ratio of studied COVID-19 CXRs (center) and performance of automatic COVID-19 diagnosis in CXR up to July 2020 (right). Performance metrics: AUC - area under the ROC curve; Acc. - accuracy; F1 - F1 score; Prec. - precision; Sens. - sensitivity; Spec. - specificity (number of works).

The scientific community has responded quickly to the challenge and numerous studies have been published in literature on automated COVID-19 diagnosis in CXR^8^. Figure 1 provides a summary in terms of the networks used, the ratio of COVID-19 vs non-COVID-19 CXRs used for developing the methods, and reported performance of automatic COVID-19 diagnosis in CXR^9^. We focus particularly on early works corresponding to initial response to the worldwide pandemic outbreak, period in which the inclusion of these type of automatic tools would have allowed to significantly reduce the workload of specialists and where high performance claims were made. Please refer for example to^9^ for a descriptive summary of these methods. As shown in Fig. 1, this is a highly imbalanced problem, with most of the datasets used in studies having less than 5% of COVID-19 images. Despite this imbalance and the high difficulty of the task, the reported performances are typically very high, commonly above 95% for different metrics.

Most methods rely on pre-trained deep networks, namely ResNet or DenseNet-based that have as main goal distinguishing COVID-19 patients from non-COVID-19 patients. Since COVID-19 shares radiological manifestations with pneumonia, a large number of methods approach this problem as a 3 class task. Other authors prefer to design dedicated networks for the task. A notable example is COVID-Net, one of the first solutions to be proposed for automatic COVID-19 screening^10^. The authors compare the performance of their method with a ResNet50 and a VGG19, showing that their solution performs better while requiring less computational complexity. In particular, an overall accuracy of 93.3% and a COVID-19 positive predictive value of 98.9% are reported. Despite Wang et al.’s success using a custom architecture, other authors have reported high performances using pre-existing architectures. For instance, Apostolopoulos et al. tested multiple CNN architectures in both 2-class (COVID-19 and not COVID-19) and 3-class (normal, pneumonia and COVID-19) scenarios, obtaining a 98.75% accuracy using a 2-class VGG19^11^ architecture^12^. Ozturk et al. proposed a variation of the DarkNet-19^13^ architecture, DarkCOVIDNet, and, similar to Apostolopoulos et al., tested both 2-class and 3-class scenarios, obtaining an accuracy of 98.08% in binary classification^14^.

Even though these results are extremely promising, the performances reported are significantly different from what has been reported for visual reading by radiologists. In Stephanie et al.^15^, 508 CXRs were interpreted by 4 radiologists and it is shown that sensitivity and specificity change as the disease progresses, from 55% to 79% sensitivity and from 83% to 70% specificity at $\le$ 2 days and > 11 days after diagnosis respectively. While it is possible for deep learning techniques to learn features that are not evaluated by radiologists, which would lead to superior performance, the dependence of these techniques on the data used for training and testing could seriously bias results. This is particularly problematic given the limited CXR COVID-19 data publicly available at the time of the worldwide outbreak and the sources from which the data was obtained. Most studies use public datasets such as the COVID-19 Image Data Collection (COVID-19 IDC)^16^ and COVIDx^10^, which were compiled by combining pre-pandemic public datasets (for normal and pathological non-COVID-19 cases) with more recent COVID-19 positive CXRs, mostly extracted from academic articles and online publications. This could cause automatic methods to learn non-radiological features such as image quality, acquisition settings and equipment, laterality or other markers, hospital system of origin, etc.. Because the positive and negative classes belong to different sources, these spurious differences highly correlate with the classes, easing the learning of shortcuts rather than actual radiological features. This was highlighted by DeGrave et al.^17^, where it was shown that a system trained on a dataset from different sources with an internal test area under the curve (AUC) of 0.992 falls to 0.76 on an external dataset.

### Contributions

The goals of this study are thus twofold: (i) to develop an automatic COVID-19 detection method in CXR to serve as a 2nd opinion to support clinical decisions in the triage of COVID-19 patients; (ii) to assess the clinical applicability of deep learning systems for COVID-19 screening using CXR images. In particular, we contribute to the development of more robust COVID-19 automatic detection methods by:

- Critically comparing the intra- and inter-dataset performance in both public and in-house datasets of a deep learning model trained following similar methods to the ones proposed during the worldwide pandemic outbreak. In particular, we show that performance claims on the literature are overconfident due to dataset bias;
- Building on the last topic, we show that using annotations from medical experts can significantly mitigate dataset bias, allowing the model to obtain a similar COVID-19 screening performance based solely on radiological manifestations of the disease to radiologists;
- To help the development and validation of future algorithms, we make publicly available the radiologists’ annotations on the public datasets.

**Figure 2:**
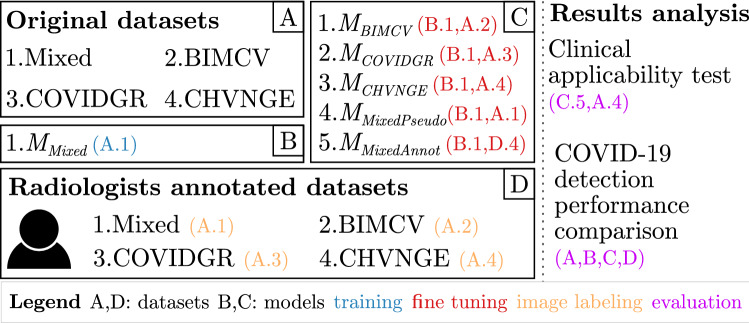
Summary of the study. Each (letter.number) indicates the data/model used for performing the task encoded by the respective color.

Figure 2 shows a summary of the study. In specific, a comparison of the performance of a ResNet-18 trained on different datasets with the annotations of two experienced radiologists is performed. It is also shown that the introduction of field-knowledge during finetuning allows to avoid the dataset bias inherent to previous solutions, improving both the system’s performance and the significance of the explanations extracted from the model. Finally, the finetuned model is tested on an external set of images aimed at replicating a clinical environment.

## Methods

### Datasets

Three public datasets and one private datasets were used in this study. All data was collected retrospectively. Note that for the public datasets, the exact criteria for inclusion in the dataset and referral for CXR are unknown. For all datasets, the same processing and criteria were applied to ensure uniformity. Only frontal CXRs—postero-anterior (PA) and antero-posterior (AP)—were included and CXRs were divided into 3 classes: *Normal*, *Pathological* (not COVID-19) and *COVID-19*. Ground truth labels for all CXRs were obtained from the ground truth available on each dataset. The *COVID-19* label corresponds to a positive SARS-CoV-2 RT-PCR result and not necessarily to the presence of radiological features of COVID-19. Table 1 shows the distribution of the number of images per dataset and class after exclusion of non-frontal CXRs. Table 2 shows the patient and CXR acquisition characteristics for each dataset after exclusion of non-frontal CXRs. This information was extracted from the metadata available for each dataset or individual DICOM metadata. Note that patient and CXR acquisition characteristics are not available for every CXR. The reader is referred to the Additional Information section for details on dataset access.

**Table 1:** Number of CXRs per dataset and (number of CXRs annotated by radiologists) for each of the three classes after exclusion of non-frontal CXRs.

Dataset	Normal	Pathological	COVID-19
Mixed			
CheXpert	21,214 (0)	169,833 (7)	0 (0)
ChestXRay-8	60,361 (188)	880 (38)	0 (0)
COVID-19 IDC	9 (1)	55 (50)	618 (205)
COVIDx	1 (0)	0 (0)	93 (4)
RSNA-PDC	8851 (251)	17,833 (291)	0 (0)
SAVE LIVES	0 (0)	0 (0)	4889 (171)
SERAM	0 (0)	5 (5)	37 (37)
Twitter	0 (0)	0 (0)	114 (8)
BIMCV			
PADCHEST	4349 (78)	9811 (170)	0 (0)
COVID-19+	0 (0)	0 (0)	2471 (41)
COVIDGR	426 (150)		426 (150)
CHVNGE	5626 (529)		735 (68)

**Table 2:** Patient and CXR acquisition characteristics for each dataset. Data is shown in absolute number and percentage in parenthesis. Age is shown as median [minimum; maximum]. * indicates that the information could not be obtained for all subjects and NA indicates that this data is not available.

	Mixed	BIMCV	COVIDGR	CHVNGE
Gender				
Male	165,123 (58.0)	8615 (51.8)	0 (0.0)	3340 (52.5)
Female	118,863 (41.7)	8015 (48.2)	0 (0.0)	3021 (47.5)
Unknown/Other	807 (0.3)	1 (0.0)	852 (100.0)	0 (0.0)
Age	58 [1;106]*	64 [1;101]*	NA	66 [2;100]
View				
AP	199,606 (70.1)	4,214 (25.3)	0 (0.0)	82 (1.3)
PA	84,070 (29.5)	12,160 (73.1)	852 (100.0)	66 (1.0)
Unknown	1117 (0.4)	257 (1.5)	0 (0.0)	6213 (97.7)
CXR equipment				
Agfa CR	2280 (0.8)	346 (2.1)	0 (0.0)	0 (0.0)
Agfa DX		247 (1.5)	0 (0.0)	0 (0.0)
Canon DX	0 (0.0)	45 (0.3)	0 (0.0)	0 (0.0)
Carestream CR	0 (0.0)	9 (0.1)	0 (0.0)	0 (0.0)
Carestream DX	0 (0.0)	97 (0.6)	0 (0.0)	59 (0.9)
FUJI CR	0 (0.0)	155 (0.9)	0 (0.0)	2164 (34.0)
FUJI DX	0 (0.0)	4 (0.0)	0 (0.0)	436 (6.9)
GE DX	0 (0.0)	155 (0.9)	0 (0.0)	0 (0.0)
GMM DX	0 (0.0)	269 (1.6)	0 (0.0)	0 (0.0)
ImagingDynamics CR	0 (0.0)	3668 (22.1)	0 (0.0)	0 (0.0)
KONICA CR	0 (0.0)	423 (2.5)	0 (0.0)	0 (0.0)
KONICA DX	0 (0.0)	89 (0.5)	0 (0.0)	0 (0.0)
Philips CR	1019 (0.4)	8131 (48.9)	0 (0.0)	0 (0.0)
Philips DX		2711 (16.3)	0 (0.0)	0 (0.0)
Samsung DX	0 (0.0)	0 (0.0)	0 (0.0)	3702 (58.2)
SIEMENS CR	1575 (0.6)	261 (1.6)	0 (0.0)	0 (0.0)
SIEMENS DX		0 (0.0)	0 (0.0)	0 (0.0)
Unknown/Other	279,919 (98.3)	21 (0.1)	852 (100.0)	0 (0.0)

#### Mixed dataset

The Mixed dataset is a combination of multiple public CXR datasets, similar to those used in most early publications on automatic COVID-19 detection in CXR^10,12,14^, combining pre-pandemic public datasets with recent COVID-19 positive CXRs, mostly extracted from academic articles and online publications. *Normal* and *Pathological* CXRs were obtained from the CheXpert^18^, ChestXRay-8^19^ and Radiological Society of North America Pneumonia Detection Challenge (RSNA-PDC)^20^ datasets. *COVID-19* positive cases and a residual amount of *Normal* and *Pathological* cases were extracted from online repositories of CXRs, namely the COVID-19 IDC^16^ and COVIDx^10^ datasets. Further CXRs were obtained by manual extraction of images published online on Twitter and the Sociedad Española de Radiologia Médica (SERAM) website. Finally, *COVID-19* positive CXR were obtained from the COVID DATA SAVE LIVES dataset, made available by the HM Hospitales.

Given that there is significant overlap in some of the datasets included in the Mixed dataset, repeated images were excluded.

#### BIMCV

The Banco digital de Imagen Medica de la Comunidad Valenciana (BIMCV) dataset is composed of CXRs from public hospitals in the Valencian Region, Spain and is made available by the BIMCV. While this dataset is composed uniquely of images from BIMCV, *Normal* and *Pathologic* (non-COVID-19) cases originate from different hospitals and from a different timespan than *COVID-19* positive cases. *Normal* and *Pathological* CXRs were obtained from the BIMCV-COVID19-PADCHEST, a subset of the larger BIMCV-PadChest^21^ public dataset which is composed of CXRs from the Hospital Universitario San Juan De Alicante, Alicante, Spain from 2009 to 2017. *COVID-19* positive CXRs were obtained from the BIMCV-COVID-19+^22^ public dataset which is composed of CXRs from 11 public hospitals in the Valencian Region, Spain from 6th February 2020 to 1st April 2020.

#### COVIDGR

The COVIDGR dataset contains CXRs from the San Cecilio University Hospital at Granada, Spain and is made available by the Andalusian Research Institute in Data Science and Computational Intelligence (DaSCI)^23^. All CXRs were acquired with the same equipment and consist entirely of PA views. All patients underwent a RT-PCR test within 24 hours of the CXR. Manual selection of CXRs was performed to balance the positive and negative RT-PCR test results. Note that because no information except for RT-PCR results is available, RT-PCR negative patients cannot be labelled exclusively as either *Normal* or *Pathological*.

#### CHVNGE

The CHVNGE dataset contains CXRs collected retrospectively at the Centro Hospitalar de Vila Nova de Gaia e Espinho (CHVNGE) in Vila Nova de Gaia, Portugal between the 21st of March and the 22nd of July of 2020. All data was acquired under approval of the CHVNGE Ethical Committee and followed all relevant guidelines and regulations. Informed consent was waived by the CHVNGE Ethical Committee given that all data was anonymised prior to any analysis. All CXRs of patients who underwent an RT-PCR test within the study time frame were extracted. Both PA and AP views were included and no other exclusion criteria were applied. The information regarding the CXR equipment used for acquisition was preserved as different CXR equipments were used in each hospital service. CXRs obtained in the emergency department (Samsung DX) can thus be analysed separately from those who were in inpatient services (Carestream DX, FUJI CR and FUJI DX) and in intensive care units (FUJI CR). CXRs from patients with a positive RT-PCR test were labelled as *COVID-19* whereas all other patients were labelled as *Normal*/*Pathological*. Note that, similarly to COVIDGR, RT-PCR negative patients cannot be labelled exclusively as either *Normal* or *Pathological*. Given that the dataset encompasses all patients who were suspected for SARS-CoV-2 infection and no manual selection of CXRs was performed, this dataset is the most representative of a clinical setting.

### CXR annotation

In order to evaluate the performance of radiologists in the detection of COVID-19 radiological features in CXR, manual annotation of a subset of CXR images from each dataset was performed by two radiologists using an in-house software. The software presented CXRs from a randomly selected subset and allowed for window center/width adjustment, zooming and panning. Radiologists were asked to label CXRs into one of 4 classes: *Normal*, *Not indicative of COVID-19 (pathological)*, *Indicative of COVID-19* and *Undetermined*. The *Indicative of COVID-19* class was defined as CXRs where the patient presented findings indicative of COVID-19, namely bilateral pulmonary opacities of low/medium density. The *Undetermined* class was defined as CXRs where the patient presented findings that could be indicative of COVID-19 but which could also be indicative of another condition, namely unilateral lung opacities, diffuse bilateral opacities of ARDS pattern or diffuse reticular opacities. The *Not indicative of COVID-19 (pathological)* class was defined as CXRs where the patient presented findings indicative of any other pathology except for COVID-19. CXRs where the patient presented medical devices were classified as *Normal* if the underlying pathology was not visible. Additionally, CXRs without sufficient quality for visual assessment by the radiologists due to bad image quality, patient positioning or any other factors could be labelled as *Compromised* for exclusion.

Manual labelling of CXRs was performed in two stages. First, both radiologists independently classified each CXR. CXRs where the two radiologists disagreed were then selected for the second stage where the two radiologists assessed the CXRs together to achieve consensus. At no point were radiologists given access to the ground truth label, RT-PCR results or any other information besides the CXR image.

To ensure that written information present in the CXR image (such as hospital system, health service, laterality markers, patient positioning, etc.) did not bias the annotation, all written labels were blacked out during before annotation. This was done in a semi-automatic way using a YOLOv3^24^ architecture for the detection of written labels in CXRs. For this purpose, 317 CXRs were randomly selected from the Mixed dataset and bounding boxes were manually drawn around all written labels. The network was then trained on the 317 CXRs (733 bounding boxes). Previous to CXR annotation by the radiologists, all CXRs were visually inspected and any missing or incorrect bounding boxes were corrected manually. All annotations on the public datasets are available at 10.25747/342B-GF87.

### Automatic CXR COVID-19 detection

For automatic COVID-19 detection in CXR, an 18-layer deep residual neural network architecture (ResNet18)^25^ was used in all experiments. While larger networks such as DenseNet121 have shown excellent performance in CXR disease classification^18^, the smaller ResNet18 was chosen to try to reduce overfitting to the limited data in the *COVID-19* class and the inherent bias of the existing datasets.

The ResNet18 architecture used was identical to the architecture proposed in He et al.^25^, except that the input is a 1-channel gray image (512 × 512 pixels) and the number of output nodes is the number of classes being considered.

The loss function used during training was the weighted binary cross-entropy^26^:

1 $$\begin{aligned} {\mathscr {L}}= & {} -\sum _{c=1}^C w_c {y_{c}} log(p_{c}) \end{aligned}$$

where *C* is the number of classes, $y_{c}$ is one when *c* is the ground truth class and $p_c$ is the model prediction for class *c*. To balance the effect of different classes and avoid bias, class weights $w_c={1-N_c/N}$ were applied, where *N* is the total number of images in the training set and $N_c$ the number of images in the training set of class *c*. Model optimization was performed using Adam^27^ with a learning rate of 0.0001 and a batch size of 24. These values were chosen empirically based on previous experiments and hardware capacity. Given the large number of images in the Mixed dataset, an epoch was defined as 1200 batches (approximately one tenth of the dataset) and all models were trained for a maximum of 100 epochs with a patience of 10 epochs as an early stopping criterion.

To estimate the performance of the predictive model in practice and avoid a possible bias due to random division of the data, a 5-fold cross-validation scheme was performed during training and testing. Folds were constructed through random CXR selection so that the class and dataset distributions in each fold are similar to the full dataset and so that all CXRs from each patient are placed in the same fold. In interdataset settings, *i.e.* when a model trained on dataset A is tested on dataset B, the full dataset B is used for testing.

## Experiments

### CXR annotation

A total of 2,442 CXRs were selected for annotation by the two radiologists. Of these, 1256 belong to the Mixed dataset, 289 belong to BIMCV, 300 belong to COVIDGR and 597 belong to CHVNGE (distribution per classes shown in Table 1). Selection of CXRs for annotation was performed randomly: for the Mixed dataset, a balanced selection strategy was used during image selection, whereas for BIMCV, COVIDGR and CHVNGE, the dataset class distribution was maintained in the subset selected for annotation. Due to practical reasons, the independent reading of CXRs by each radiologist was not possible in all cases—of the 2,442 CXRs annotated, 799 were annotated in consensus without independent reading.

During annotation, a total of 77 CXRs (25 *Normal*, 26 *Pathological* and 26 *COVID-19*) were randomly selected for repeated annotation to determine intraobserver variability. The repeated images were mixed in with new images in a 1:10 ratio during annotation. Radiologists were unaware that repeated images were being introduced to avoid bias.

### Model training

#### Baseline training

The network was first initialized with weights from an Imagenet pretrained model^28^ (the weights in the first layer were taken from the red channel on the pretrained model). The model was first trained for binary *Normal* vs *Not Normal* classification and only then trained with three output nodes corresponding to the *Normal*, *Pathological* and *COVID-19* classes where the *Pathological* class included all classes except the *Normal* and *COVID-19* classes. This two-step training strategy aims at leading the model to learn CXR-related features prior to learning COVID-19-related features, increasing feature relevance while reducing overfitting. This model was trained using the Mixed dataset to allow a direct comparison to previous studies on COVID-19 detection in CXR and will be referred to as $M_{Mixed}$.

#### Dataset finetuning

In order to estimate a best case scenario in terms of performance for each dataset, $M_{Mixed}$ was then retrained on each of the other single-source datasets—BIMCV, COVIDGR and CHVNGE. By having access to data from each dataset, dataset-specific features can be learned, improving performance. Naturally, finetuned models will also be more subject to dataset bias and learning shortcuts that misrepresent COVID-19 manifestations. These models are hereinafter referred to as $M_{BIMCV}$, $M_{COVIDGR}$ and $M_{CHVNGE}$ respectively.

#### Pseudo-labelling

Given the characteristics of the Mixed dataset and the high correlation between dataset sources and classes, it is expected that $M_{Mixed}$ model learns not only radiological features of COVID-19 but also that it relies heavily on learning shortcuts^17^. In order to decrease this effect and improve the learning of radiological features, a pseudolabelling strategy^29^ was implemented. This was performed by obtaining the predictions of the model trained on the Mixed dataset on all images of the same dataset. All images with predicted probability lower than 0.9 were then removed to include only high confidence predictions and the predicted class was then used as ground truth label for retraining the model. This model is referred as $M_{MPseudo}$.

#### Radiologist annotations

Given that not all COVID-19 positive cases present manifestations on CXR, considering these cases as *COVID-19* during training can introduce high levels of noise and promote the learning of shortcuts for classification, which do not represent COVID-19 manifestations. Using radiologist annotations during training avoids this issue as COVID-19 positive CXRs without manifestations will be presented to the model as *Normal* CXRs during training, enforcing the learning of features that represent COVID-19 manifestations. For this purpose, $M_{Mixed}$ was retrained using the labels given by the radiologists during manual annotation of the Mixed dataset. Regarding the CXRs annotated as *Undetermined*, the principle of precaution was applied: given the definition of this class as CXRs where the patient presented findings that could be indicative of COVID-19 but which could also be indicative of another condition, these CXR were considered to belong to the *COVID-19* class. CXRs marked as Compromised were discarded. This model is hereinafter referred to as $M_{MAnnot}$.

### Performance evaluation

The agreement between radiologist annotations and ground truth labels in the detection of COVID-19 was evaluated in terms of precision (Prec.) and recall whereas the intra- and interobserver variability were evaluated in terms of accuracy (Acc.) and Cohen’s kappa ($\kappa$)^30^:

2 $$\begin{aligned} \text {Recall}= & {} \frac{TP}{TP+FN} \end{aligned}$$

3 $$\begin{aligned} \text {Prec.}= & {} \frac{TP}{TP+FP} \end{aligned}$$

4 $$\begin{aligned} \text {Acc.}= & {} \frac{TP + TN}{TP + TN + FP + FN} \end{aligned}$$

5 $$\begin{aligned} \kappa= & {} 1-\frac{1-p_o}{1-p_e} \end{aligned}$$

where *TP* is the number of true-positive detections, *FN* the number of false-negatives, *FP* the number of false-positives and *FN* is the number of false-negatives. $p_o$ is the relative agreement between annotators and $p_e$ is the expected agreement when both annotators assign labels randomly. All metrics were computed considering a binary classification scenario (*COVID-19* or not *COVID-19*). Given the definition of the *Undetermined* class, agreement was computed considering as positives either only the *Indicative of COVID-19* class (C) or both the *Indicative of COVID-19* and *Undetermined* classes (C+U). Where applicable, the statistical significance of the difference between the radiologist annotations and the ground truth labels was tested using an adaptation of the McNemar test ($\chi ^2$)^31^. McNemar tests were computed only when power $\ge \!0.8$ and statistical significance is reported in terms of p-value.

Model performance was evaluated with receiver operating characteristic (ROC) curve, specifically in terms of AUC. Confidence intervals were computed taking into account the achieved average performance for all folds. To further validate the models, Grad-CAM++^32^ was used to visualize the location of the regions responsible for the network predictions when necessary. Finally, the calibration of the model’s prediction was assessed using the Expected Calibration Error (ECE)^33^. The ECE is a summary of the Reliability diagram, which plots the expected accuracy as function of the predicted class probability. Briefly, all *N* test samples are binned probability-wise in *B* groups for which the accuracy $\text {Acc}_b$ and average confidence ${\overline{p}}_b$ for the corresponding reference label are computed. ECE is the weighted average of the difference between these two measures:

6 $$\begin{aligned} \text {ECE}= & {} \sum _{b=1}^B \frac{N_b}{N} |\text {Acc}_b - {\overline{p}}_b| \end{aligned}$$

where $N_b$ is the number of samples in bin *b*. Results are evaluated considering 10 bins.

The statistical significance of the differences in the performance of the models (and the radiologists) was performed according to the DeLong test, which allows for paired comparison of AUCs^34^. When comparing AUCs across different test sets, the permutation test for continuous unpaired comparison of AUCs proposed in Venkatraman et al.^35^ was used. Note that when comparing a model to radiologist annotations, only the subset of CXRs annotated by radiologists was taken into account. Statistical significances computed across folds were fused according to Fisher’s combined probability test^36^ to obtain a single p-value and when performing multiple comparisons, statistical significance was considered after applying the Bonferroni correction^37^.

## Results

### CXR annotation

**Figure 3:**
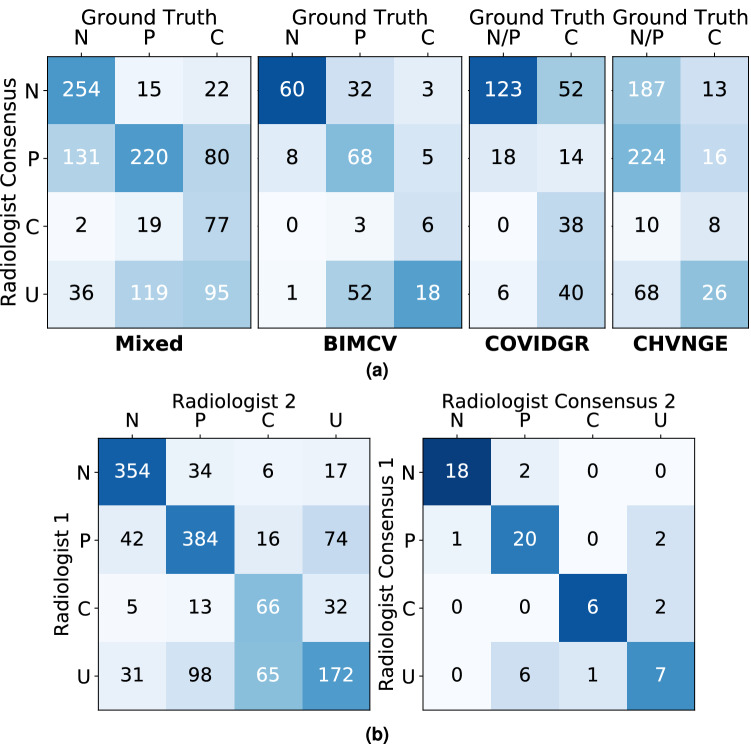
Confusion matrices between ground truth labels and radiologist annotations. (**a**) Comparison between ground truth and radiologists’ consensus on each dataset; (**b**) Inter- and intraobserver variability across all datasets (left and right respectively). N - *Normal*; P - *Not indicative of COVID-19 (pathological)*; C - *Indicative of COVID-19*; U - *Undetermined*. Cases annotated as Compromised are not shown. Color intensity corresponds to the percentage of cases within each column.

Figure 3 shows the confusion matrices between ground truth labels and radiologist annotations in each of the datasets, as well as interobserver variability and intraobserver variability. The performance of radiologists in COVID-19 detection in terms of precision and recall is shown in Table 3. As expected, considering as positives only CXRs marked as *Indicative of COVID-19* (C) gives a higher average precision but with low recall, whereas including as positives CXRs marked as *Undetermined* (C + U) significantly increases recall, but at the expense of precision. Comparing across datasets, it can be seen that radiologists achieve the highest precision and recall on the COVIDGR and Mixed datasets, whereas the lowest performance is obtained for the CHVNGE dataset. Table 4 shows the inter- and intraobserver variabilities for all annotated CXRs. A statistically significant difference ($p\,=\,0.0021$) was found between the two observers when considering as positives only the CXRs marked as *Indicative of COVID-19*, whereas other combinations could not be performed at power $\ge \!0.8$ due to the reduced sample size.

**Table 3:** Agreement between radiologists after consensus and ground truth considering as positives only CXRs marked as *Indicative of COVID-19* (C) or *Indicative of COVID-19* and *Undetermined* (C + U). $\chi ^2$: p-value obtained with the McNemar test.

Dataset	C			C + U		
	Prec.	Recall	$\chi ^2$	Prec.	Recall	$\chi ^2$
Mixed	0.79	0.28	< 0.0001	0.49	0.63	< 0.0001
BIMCV	0.67	0.19	< 0.0001	0.30	0.75	< 0.0001
COVIDGR	1.00	0.26	< 0.0001	0.93	0.54	< 0.0001
CHVNGE	0.44	0.13	< 0.0001	0.30	0.54	< 0.0001
All	0.79	0.25	< 0.0001	0.49	0.60	< 0.0001

**Table 4:** Inter- and intraobserver variability of radiologist annotations considering as positives only CXRs marked as *Indicative of COVID-19* (C) or *Indicative of COVID-19* and *Undetermined* (C + U). $\chi ^2$: p-value obtained with the McNemar test (NP: power$<0.8$).

	C			C + U		
	Acc.	$\kappa$	$\chi ^2$	Acc.	$\kappa$	$\chi ^2$
Interobserver	0.90	0.44	0.0021	0.82	0.58	NP
Intraobserver						
Radiologist 1	0.96	0.80	NP	0.81	0.56	NP
Radiologist 2	0.87	0.50	NP	0.83	0.63	NP
Consensus	0.95	0.77	NP	0.88	0.71	NP

### Automatic CXR COVID-19 detection

**Figure 4:**
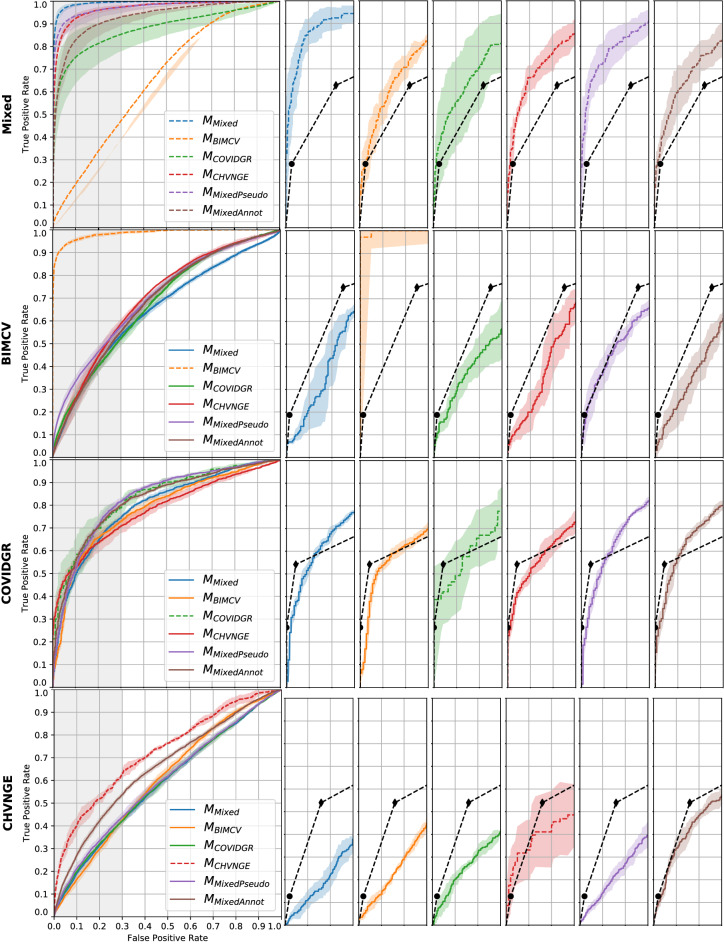
ROC of each of the models on each of the datasets. Average model performance across all folds is shown as a line (full line: full dataset used for testing; dashed line: separate test set for each fold) and shaded region corresponds to the 95% confidence interval. Left: model performance on all CXRs; Right: model and radiologists performance on annotated CXRs. Rightmost plots are a zoomed version of the gray shaded region of the left plot. Radiologists performance is shown when considering as positives only CXRs marked as *Indicative of COVID-19* ($\bullet$) or *Indicative of COVID-19* and *Undetermined* ($\blacklozenge$).

Figure 4 shows the ROC curves of all trained models on each dataset, as well as comparison to radiologist annotations after consensus. Table 5 shows the AUC of each model in all datasets and Table 6 shows the statistical significance of differences in AUC between readers (trained models and radiologists) for each dataset. Table 7 shows the model calibration metric ECE for each dataset, model and fold.

On the test set, it can be seen that intradataset train-test scenarios obtained the best results on all datasets, i.e. when training includes CXRs from the dataset used on testing (dashed lines in Fig. 4). This was most evident on the Mixed, BIMCV and CHVNGE datasets with differences in AUC between $M_{Mixed}$ and other models on the Mixed dataset, between $M_{BIMCV}$ and other models on the BIMCV dataset and between $M_{CHVNGE}$ and other models on the CHVNGE dataset all being statistically significant with $p\,<\,0.0001$. On COVIDGR, differences in ROCs were less stark. The differences between $M_{COVIDGR}$ and $M_{Mixed}$ and $M_{COVIDGR}$ were statistically significant for $p\,<\,0.0015$ but differences between $M_{COVIDGR}$ and other models were less significant.

On the subset of annotated CXRs, trained models outperformed radiologists on intradataset train-test scenarios, particularly on the Mixed and BIMCV datasets. Differences in performance between radiologists and each of the models yielded statistically significant differences ($p\,<\,0.0001$) for all models on the Mixed dataset and for $M_{BIMCV}$ on the BIMCV dataset. On COVIDGR and CHVNGE, differences between radiologists and $M_{COVIDGR}$ and $M_{CHVNGE}$ were less significant ($p\,=\,0.0137$ and $p\,=\,0.03691$). On interdataset train-test, model performance is typically lower than or similar to that of radiologists. On CHVNGE, where model performance was lowest, only $M_{MAnnot}$ can achieve a performance close to that of radiologists.

**Table 5:** Model AUC for each dataset and cross validation fold. Bold indicates the highest AUC per dataset and fold.

	$M_{Mixed}$	$M_{BIMCV}$	$M_{COVIDGR}$	$M_{CHVNGE}$	$M_{MPseudo}$	$M_{MAnnot}$
Mixed	**0.9967**	0.6248	0.9294	0.9741	0.9890	0.9192
	**0.9898**	0.7161	0.9144	0.9590	0.9326	0.9017
	**0.9892**	0.7139	0.7708	0.9604	0.9785	0.9007
	**0.9906**	0.6072	0.8275	0.9783	0.9840	0.9694
	**0.9974**	0.6078	0.9535	0.9718	0.9853	0.9635
BIMCV	0.6354	**0.9790**	0.6572	0.6929	0.7357	0.6264
	0.6564	**0.9839**	0.6659	0.7005	0.6887	0.6813
	0.6405	**0.9877**	0.6510	0.7009	0.6952	0.6858
	0.6491	**0.9867**	0.6861	0.6861	0.7160	0.7249
	0.6399	**0.9847**	0.6815	0.6965	0.6755	0.6740
COVIDGR	0.8441	0.8272	0.8409	0.7400	0.8384	**0.8789**
	0.7802	0.6768	**0.8789**	0.7868	0.8060	0.7774
	0.8065	0.8180	0.7936	0.7697	0.8209	**0.8424**
	0.7871	0.8215	0.8143	0.7701	0.8004	**0.8611**
	0.7432	0.7116	0.8227	0.7991	**0.8590**	0.7625
CHVNGE	0.5926	0.6244	0.5730	**0.7218**	0.5810	0.6986
	0.6085	0.6025	0.5971	**0.7145**	0.5969	0.6518
	0.5549	0.6178	0.5971	**0.7322**	0.5887	0.6137
	0.5465	0.5891	0.6292	**0.7269**	0.6049	0.6604
	0.6256	0.5674	0.5573	**0.7293**	0.6297	0.6594

**Table 6:** Statistical significance of differences in AUC between readers (models and radiologists) for each dataset according to the De Long test. Bold indicates statistical significance with Bonferroni correction ($p\,<\,0.0033$ and $p\,<\,0.0024$ for the test set and annotated test sets respectively).

		Whole test set				Annotated test set			
		Mixed	BIMCV	COVIDGR	CHVNGE	Mixed	BIMCV	COVIDGR	CHVNGE
$M_{Mixed}$	$M_{BIMCV}$	< **0.0001**	< **0.0001**	0.6026	0.0481	< **0.0001**	< **0.0001**	0.2155	0.0376
$M_{Mixed}$	$M_{COVIDGR}$	< **0.0001**	< **0.0001**	**0.0013**	0.2237	< **0.0001**	0.9545	0.6534	0.5636
$M_{Mixed}$	$M_{CHVNGE}$	< **0.0001**	< **0.0001**	0.1189	< **0.0001**	<**0.0001**	0.3035	0.5661	**0.0023**
$M_{Mixed}$	$M_{MPseudo}$	< **0.0001**	< **0.0001**	**0.0001**	0.1163	**0.0005**	< **0.0001**	0.7054	0.4333
$M_{Mixed}$	$M_{MAnnot}$	< **0.0001**	< **0.0001**	0.1080	< **0.0001**	**0.0000**	0.5516	0.7556	0.1077
$M_{BIMCV}$	$M_{COVIDGR}$	< **0.0001**	< **0.0001**	0.0102	0.0613	**0.0000**	< **0.0001**	0.2324	0.0119
$M_{BIMCV}$	$M_{CHVNGE}$	< **0.0001**	< **0.0001**	0.6802	< **0.0001**	0.3088	< **0.0001**	0.5906	0.2749
$M_{BIMCV}$	$M_{MPseudo}$	< **0.0001**	< **0.0001**	0.0319	0.1726	**0.0000**	< **0.0001**	0.0813	0.0125
$M_{BIMCV}$	$M_{MAnnot}$	< **0.0001**	< **0.0001**	0.2073	**0.0013**	0.0425	< **0.0001**	0.0433	0.0957
$M_{COVIDGR}$	$M_{CHVNGE}$	< **0.0001**	**0.0001**	**0.0015**	< **0.0001**	< **0.0001**	0.7269	0.1279	**0.0003**
$M_{COVIDGR}$	$M_{MPseudo}$	< **0.0001**	< **0.0001**	0.2827	0.0139	< **0.0001**	0.1835	0.6108	0.6498
$M_{COVIDGR}$	$M_{MAnnot}$	< **0.0001**	0.0277	0.0055	< **0.0001**	< **0.0001**	0.8138	0.1589	0.1793
$M_{CHVNGE}$	$M_{MPseudo}$	< **0.0001**	< **0.0001**	0.0067	< **0.0001**	0.0127	0.0893	0.2426	0.0070
$M_{CHVNGE}$	$M_{MAnnot}$	< **0.0001**	**0.0002**	0.0059	< **0.0001**	0.0234	0.7022	0.3261	0.0528
$M_{MPseudo}$	$M_{MAnnot}$	< **0.0001**	< **0.0001**	0.2475	< **0.0001**	**0.0003**	0.3714	0.4898	0.1156
Radiologists	$M_{Mixed}$	-	-	-	-	< **0.0001**	0.0594	0.2566	0.0028
Radiologists	$M_{BIMCV}$	-	-	-	-	< **0.0001**	< **0.0001**	0.7046	0.1386
Radiologists	$M_{COVIDGR}$	-	-	-	-	< **0.0001**	0.3913	0.0137	0.0092
Radiologists	$M_{CHVNGE}$	-	-	-	-	< **0.0001**	0.2694	0.7197	0.3691
Radiologists	$M_{MPseudo}$	-	-	-	-	< **0.0001**	0.4794	0.0331	0.0212
Radiologists	$M_{MAnnot}$	-	-	-	-	< **0.0001**	0.0053	0.1322	0.2046

**Figure 5:**
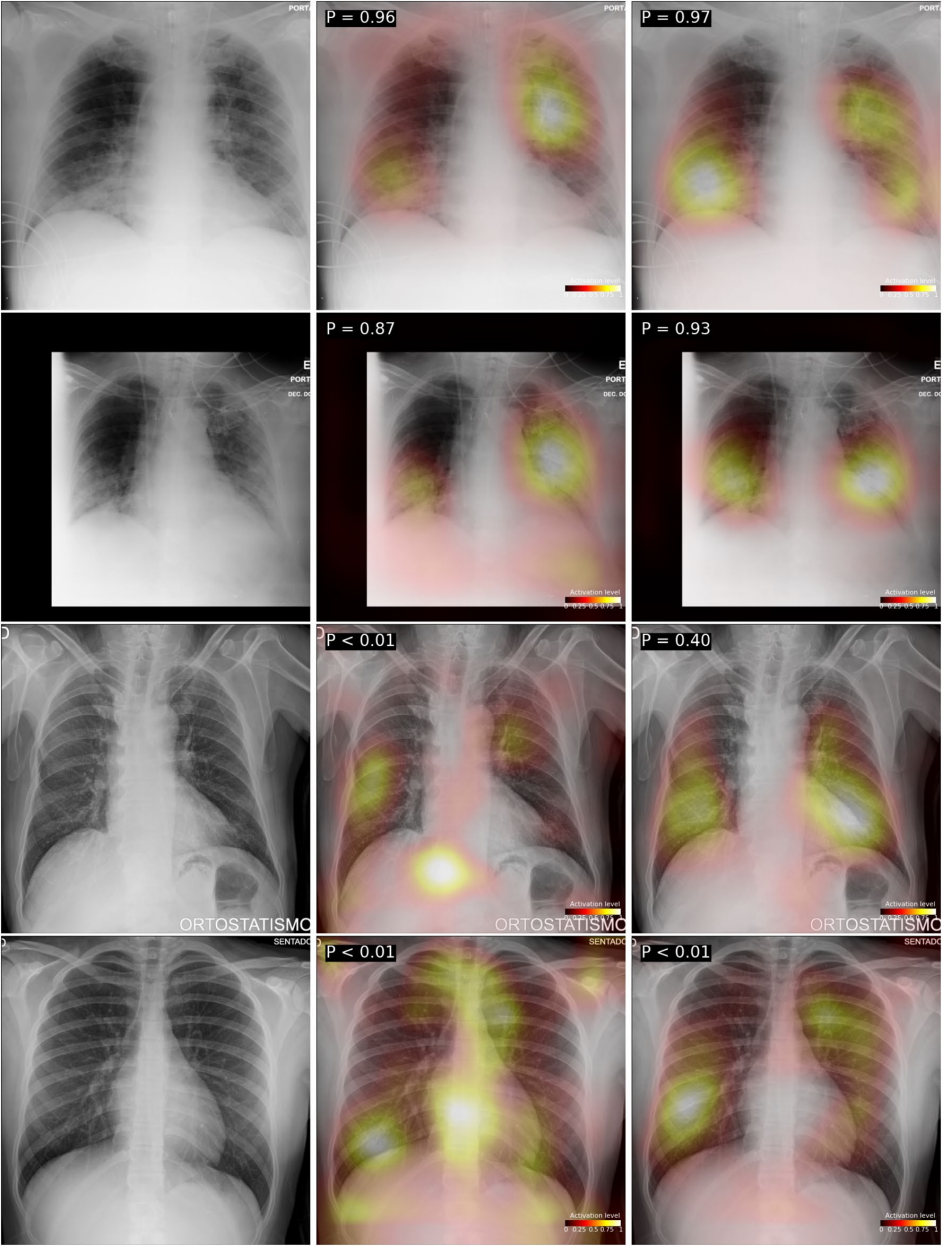
Examples of CXRs of *COVID-19* positive patients from the CHVNGE dataset (first column) and corresponding GradCAM++ activation on the *COVID-19* class for the $M_{Mixed}$ (second column) and the $M_{MAnnot}$ (third column). The first two rows correspond to CXRs correctly classified by $M_{MAnnot}$ with high probability, the third row corresponds to a CXR correctly classified by $M_{Mixed Annot}$ with average probability and the fourth row corresponds to a CXR incorrectly classified by $M_{Mixed Annot}$ with low probability.

Figure 5 shows the GradCAM++ activations of $M_{Mixed}$ and $M_{MAnnot}$ in CXRs of *COVID-19* positive patients from the CHVNGE dataset. Images were selected from representative subsets containing the 10 CXRs with predicted probability closest to the maximum, average and minimum predicted probability by $M_{MAnnot}$.

**Table 7:** Calibration in termos of ECE for each model and dataset for the 5 test folds. Lower ECE values indicate higher calibration. Bold indicates lowest value in row.

	$M_{Mixed}$	$M_{BIMCV}$	$M_{COVIDGR}$	$M_{CHVNGE}$	$M_{MPseudo}$	$M_{MAnnot}$
Mixed	0.0082	0.6994	0.0883	0.0448	**0.0079**	0.0438
	**0.0191**	0.6977	0.1161	0.0904	0.0224	0.0265
	**0.0215**	0.6021	0.2232	0.0291	0.0242	0.0245
	**0.0213**	0.7106	0.0648	0.0498	0.0294	0.0243
	0.0241	0.7152	0.0633	0.0217	0.0251	**0.0104**
BIMCV	0.1469	**0.0069**	0.1992	0.1145	0.1234	0.1639
	0.1304	0.2277	0.1326	**0.0670**	0.1442	0.0929
	**0.1202**	0.2074	0.2724	0.1535	0.1255	0.1350
	0.1295	0.2237	0.1555	**0.0307**	0.1194	0.1052
	0.1382	0.2209	0.2260	**0.0971**	0.1334	0.1489
COVIDGR	0.4447	0.2275	**0.0810**	0.1029	0.4238	0.1902
	0.4591	0.1790	0.1961	**0.0773**	0.4977	0.2853
	0.3522	0.1970	0.2810	**0.0909**	0.4321	0.2300
	0.4344	0.2442	0.3393	**0.0419**	0.4055	0.2572
	0.4373	0.2008	0.3581	**0.1109**	0.4595	0.2026
CHVNGE	0.1202	0.6024	0.1281	0.0650	0.1039	**0.0599**
	0.0965	0.5253	0.1523	**0.0349**	0.1136	0.0552
	0.0941	0.5427	0.2279	0.0913	0.1131	**0.0790**
	0.0990	0.6492	0.1636	0.0815	0.1447	**0.0504**
	0.1046	0.6215	0.2241	0.0779	0.1075	**0.0635**

#### Real-world application

**Figure 6:**
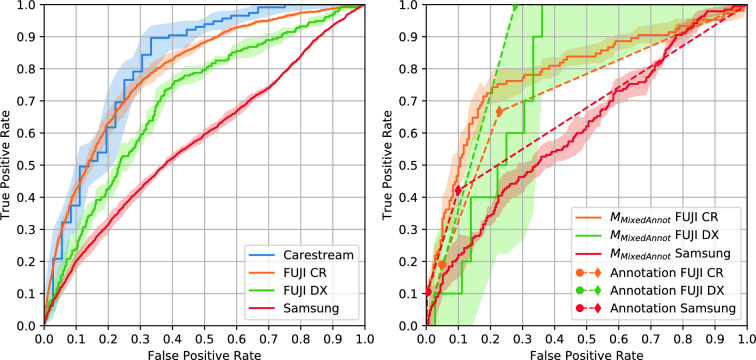
Equipment-wise evaluation of $M_{MAnnot}$ on the CHVNGE dataset. Top left: model performance on all CXRs. Top right: model and radiologist performance on CXRs annotated by radiologists (Carestream not shown due to limited data). Average model performance for all folds is shown as a line and shaded region corresponds to the 95% confidence interval. Radiologists’ performance is shown considering as positives only CXRs marked as *Indicative of COVID-19* ($\bullet$) or *Indicative of COVID-19* and *Undetermined* ($\blacklozenge$).

Figure 6 shows the performance of $M_{MAnnot}$ and the radiologist annotations for each CXR equipment used in acquisition on CHVNGE, which correspond to the different hospital services as outlined in Section “Datasets”. Table 8 shows the AUC for every cross validation fold for each CXR equipment. $M_{MAnnot}$ was chosen for this analysis as it was the best performing method on CHVNGE, excluding the model finetuned on CHVNGE. Table 9 shows the statistical significance of differences in $M_{MAnnot}$ AUC between CXR equipments. Table 10 shows the statistical differences in ROC between $M_{MAnnot}$ and radiologists on the subset of annotated CXRs. It can be seen that performance is higher for CXRs acquired with Carestream and FUJI CR, followed by FUJI DX. The lowest AUC is obtained for CXRs obtained with Samsung with a statistical significant difference to FUJI CR and Carestream ($p\,<\,0.0046$). The performance of radiologists follows the same trend as $M_{MAnnot}$, with the lowest sensitivity found for Samsung CXRs. Nevertheless, radiologists showed a significantly superior AUC for Samsung CXRs ($p\,=\,0.0398$) and significantly inferior performance on FUJI CR CXRs ($p\,=\,0.0415$).

**Table 8:** $M_{MAnnot}$ AUC for each CXR equipment on CHVNGE for each cross validation fold. Bold indicates the highest AUC for each fold.

FUJI CR	Samsung	Carestream	FUJI DX
0.7808	0.5618	**0.8056**	0.6768
0.8145	0.5710	**0.8551**	0.7053
0.7770	0.5676	**0.7959**	0.7061
0.7908	0.5934	**0.8249**	0.7291
0.7975	0.5890	**0.8019**	0.6783

**Table 9:** Statistical significance of differences in $M_{MAnnot}$ AUC between different CXR equipments on CHVNGE according to the Venkatraman test. Bold indicates statistical significance with Bonferroni correction ($p\,<\,0.0083$).

		$$M_{MAnnot}$$
FUJI CR	Samsung	< **0.0001**
FUJI CR	Carestream	1.0000
FUJI CR	FUJI DX	0.2357
Samsung	Carestream	**0.0046**
Samsung	FUJI DX	0.0294
Carestream	FUJI DX	0.8490

**Table 10:** Statistical significance of differences in ROC between $M_{MAnnot}$ and radiologists for each CXR equipment on CHVNGE according to the De Long test. Bold indicates $p\!<\!0.05$.

		FUJI CR	Samsung	FUJI DX
$M_{MAnnot}$	Radiologists	**0.0415**	**0.0398**	0.2699

## Discussion

### CXR annotation

The radiologists’ recall for *Indicative of COVID-19* and *Undetermined* is in line with other studies^15^. On the other hand, the recall for *Indicative of COVID-19* is lower, suggesting that the *Indicative of COVID-19* labelling protocol is overly conservative as it only includes CXRs where radiologists were fairly certain that the patient presented COVID-19 infection. Regarding precision, the differences observed between datasets are likely related to the characteristics of each dataset. It can be seen that on both the Mixed and BIMCV datasets, false positive COVID-19 annotations mostly occur for pathological non-COVID-19 cases (Fig. 3a) and rarely occur for normal patients. As such, for datasets where images originate from multiple sources and represent a wider range of pathologies confoundable with COVID-19, precision is lower. While the *Normal*/*Pathological* distribution is not known for COVIDGR, 82% of non-*COVID-19* cases were annotated as *Normal* by the radiologists, which indicates that the percentage of *Pathological* cases is significantly lower than on other datasets and is responsible by the high precision values obtained. Furthermore, it shows how dataset characteristics can bias the performance obtained by radiologists but also for models being tested on these datasets.

Interestingly, the radiologists’ performance on the CHVNGE dataset is lower than for the other datasets. This suggests that the CHVNGE dataset is more challenging and that the public datasets misrepresent the different COVID-19 stages in comparison to the clinical reality in CHVNGE. This is particularly true for the Mixed dataset, which is known to mostly include severe COVID-19 patients^23^. On the other hand, CHVNGE may present a higher prevalence of early stage COVID-19 cases, which have limited radiological manifestations, resulting in lower recall. This hypothesis is corroborated by Fig. 6, where the experts’ performance on images acquired in inpatient services and the intensive care unit is higher than on images acquired on initial patient screening in the emergency department.

Regarding the inter- and intraobserver variability, high accuracy ($\ge$0.81) was obtained with however only moderate $\kappa$ values, particularly for interobserver variability when considering as positives C. Analysing Fig. 3b, it can be seen that while negative *Indicative of COVID-19* CXR annotations are consistent, CXRs annotated by at least one of the radiologists as *Indicative of COVID-19* are much less consistent, with radiologist 1 being in general more conservative and attributing label *Undetermined* to a high proportion of CXRs annotated as *Indicative of COVID-19* by radiologist 2. This difference in agreement is however, expected since the *Undetermined* label corresponds to borderline cases, where the main radiological manifestations of COVID-19 are not obvious or complete. Consequently, decisions in these cases may vary more frequently. This lack of consistency is however less clear when considering as positives C + U, where a reasonably higher value of $\kappa$ is obtained.

### Automatic CXR COVID-19 detection

The AUC differences found between inter- and intradataset train-test scenarios (Table 5) corroborate the findings of DeGrave et al.^17^. Even though experiments were designed with patient-wise stratified cross-validation, the performance of the models in intradataset train-test scenarios was always higher than when different datasets were used for training and testing. This suggests that the deep learning system is not relying exclusively on radiological features to perform image classification, and is instead partially overfitting to other acquisition details. This further highlights the need to carefully validate systems prior to announcing (near-)human performance. On the other hand, the studied finetuning approaches helped to mitigate the overfit behaviour. Indeed, results suggest that revisiting cases where COVID-19 radiological manifestations are more evident helps the model converge to a feature representation that better encodes the radiological manifestations of the pathology. In fact, both $M_{MPseudo}$ and $M_{MAnnot}$ are able to outperform $M_{Mixed}$ in all external datasets (Table 5), without requiring additional training data. The difference in performance in CHVNGE is particularly significant for $M_{MAnnot}$ ($p\,<\,0.0001$), where the annotations performed by the radiologists were used to finetune the system. As shown in Fig. 4, training with a selection of images from the Mixed dataset known to contain COVID-19 features allowed the model to approximate its performance to the human experts on the CHVNGE dataset without the need to introduce images from that dataset.

The hypothesis that finetuning with good quality labels improves the system’s reliability is further supported by the models’ calibration performance (Table 7). Interestingly, the lowest ECE values are achieved when using finetuning with the CHVNGE dataset ($M_{CHVNGE}$) and using the expert annotations ($M_{MAnnot}$). As previously discussed, the CHVNGE dataset may better represent the clinical reality and thus have a higher diversity and progression stages of COVID-19 radiological manifestations, promoting a less binarized output of the model. Likewise, $M_{MAnnot}$ shows low ECE values for all datasets. This further corroborates that adjusting the model’s weights using the CXRs annotated by the radiologists mitigates overfitting by redefining the solution space and allowing to dampen previously overconfident incorrect predictions.

Finally, comparing the activation maps of $M_{Mixed}$ and $M_{MAnnot}$ corroborates that the features encoded by $M_{MAnnot}$ can better represent findings indicative of COVID-19. On the two top examples, $M_{Mixed}$ activation maps fail to indicate the full extent of the lung findings, namely on the bottom left lung in the first example and present lower activations on the right lung in the second example. The activation maps also reveal that the focus of $M_{MAnnot}$ on the region of interest (i.e. the lung volume) was significantly improved when compared to $M_{Mixed}$. This can be seen not only for correct classifications, such as the second example where $M_{Mixed}$ shows activations on the diaphragm, but also for wrong classifications such as the fourth example where the strongest activations of $M_{MAnnot}$ are limited to the lung region whereas $M_{Mixed}$ presents strong activations on the heart, clavicles and diaphragm.

#### Real-world application

The performance of both $M_{MAnnot}$ and the radiologists (*Indicative of COVID-19* and *Undetermined*) is higher in images from intensive care units and inpatient services in comparison to the emergency department (Fig. 6). As previously suggested, images from patients with late stage COVID-19 are expected to be easier to distinguish from other pathologies because the radiological manifestations resulting from the infection are more visible. This further reinforces the need to contextualize the acquisition setting when reporting model performances. While it has been repeatedly suggested in literature that a system such as the one proposed in this study could be used as an early screening tool, it is clear in this study that even $M_{MAnnot}$, the best performing model in CHVNGE without dataset finetuning, has a much lower performance than what has been suggested in literature and than what can be ascertained from available public datasets where significantly higher AUCs have been reported (AUCs $\ge 0.9$). Instead, systems such as this are perhaps put to better use as an evaluation tool of the progress of severe COVID-19 infections, reducing the workload of intensivists and radiologists on intensive care units by providing an objective opinion of the disease’s progression.

### Main findings and limitations

As discussed in the Introduction section, several methods for automatic COVID-19 diagnosis in CXR have been proposed in literature. Particularly in the beginning of the pandemic extremely high performances have also been reported (see Fig. 1)^9^. These results have been replicated in this study on intradataset train-test scenarios (e.g. $M_{Mixed}$ tested on the Mixed dataset). However performance in interdataset train-test scenarios was found to be much lower, likely due to significant dataset bias. In these scenarios, most trained models could not achieve the performance of radiologists, particularly on the CHVNGE dataset, which more closely represents clinical reality. When finetuned with radiologist annotations however, $M_{MAnnot}$ showed a more consistent performance across datasets and a much closer ROC to that of radiologists on CHVNGE. Finally, performance on different hospital services (through CXR equipments) was studied, showing that the performance of an automatic system for COVID-19 detection in CXR is nevertheless underwhelming and research efforts should be directed towards, for example, evaluation of progress and severity of disease in inpatient/intensive care units.

In spite of the promising results obtained in this study, there are limitations that must be taken into account in the interpretation of results and future directions. As highlighted in this manuscript, the training of deep learning systems relies heavily on the available data and while this study includes data from several sources to achieve a good representation of multiple environments, the dataset which intends to represent clinical reality is limited. For one, it represents the reality of a single hospital system in Portugal, which may limit the reproducibility of these results in other hospitals. Although we believe that the performance differences reported on Table 8 are meaningful and justifiable, it would still be of interest to corroborate our findings on additional data sources. Furthermore, the data from CHVNGE represents a limited scope in time, which is particularly relevant given the rapid changes of COVID-19 since its appearance. As such, to properly evaluate the true clinical impact of this type of systems, different time points would need to be considered. Finally, there is no guarantee that the achieved performance and model behaviour is reproducible for different network architectures. Indeed, in this study we opted for using a ResNet architecture which, as highlighted in Fig. 1, accounts for approximately 25% of the proposed methods during the initial outbreak. However, different network architectures may have different generalization capabilities and robustness to overfit. Given the impossibility of assessing all available architectures, we aim at raising awareness for the need to properly train and evaluate a model’s performance and thus avoid overconfident claims.

## Conclusion

This study assessed the performance of a deep learning system for COVID-19 screening using CXR and compared it with expert radiologists. The detection of COVID-19 in CXR images is non-trivial due to the wide range of radiological manifestations associated with the infection. Consequently, radiologists tend to confound COVID-19 patients with other pathologies. Similarly to other recent studies, it was found that the performance reported for deep learning approaches is overconfident. Indeed, this study shows that the screening performance is not robust to changes in data origin. However, the results shown suggest that finetuning the model with labels provided by human experts allows the network to improve the quality and meaningfulness of the extracted features, improving explainability and reducing data bias.

Results also suggest that the applicability of these systems for initial patient triage, when radiological manifestations of COVID-19 are minimal, is limited. However, when radiological manifestations of COVID-19 are present, these can be accurately detected and pinpointed by these tools. Although the achieved results are promising, there is still need to understand how well these findings translate to other time points/variants of COVID-19 and different clinical realities. Based on this study, future directions in this field should also focus on the use of deep learning systems for tracking the evolution of mild to severe COVID-19 infections, providing a robust 2nd opinion and thus contributing to mitigate the consequences of the pandemic.

### Data availability

The public datasets used in this study are available in the following repositories:

- **CheXpert:** https://stanfordmlgroup.github.io/competitions/chexpert/
- **ChestXRay-8:** https://nihcc.app.box.com/v/ChestXray-NIHCC
- **COVID-19 IDC:** https://github.com/ieee8023/covid-chestxray-dataset
- **COVIDx:** https://github.com/lindawangg/COVID-Net
- **RSNA-PDC:** https://www.kaggle.com/c/rsna-pneumonia-detection-challenge
- **SAVE LIVES:** https://www.hmhospitales.com/coronavirus/covid-data-save-lives
- **SERAM:** https://seram.es/images/site/TUTORIAL_CSI_RX_TORAX_COVID-19_vs_4.0.pdf
- **Twitter:** https://twitter.com/ChestImaging
- **BIMCV PADCHEST:** https://bimcv.cipf.es/bimcv-projects/padchest/
- **BIMCV COVID-19-PADCHES:** https://bimcv.cipf.es/bimcv-projects/bimcv-covid19/
- **COVIDGR:** https://dasci.es/transferencia/open-data/covidgr-2/

Manual annotations by the radiologists on the public datasets are publicly available at 10.25747/342B-GF87. The CHVNGE dataset was acquired under approval of the CHVNGE Ethical Committee as detailed in Section“Datasets”. However, the CHVNGE Ethical Committee determined that the data cannot be used beyond the purpose of the current study, and thus cannot be shared publicly with other institutions.
